# Anti-Inflammatory and Histological Analysis of Skin Wound Healing through Topical Application of Mexican Propolis

**DOI:** 10.3390/ijms241411831

**Published:** 2023-07-23

**Authors:** Daniela Balderas-Cordero, Octavio Canales-Alvarez, Roberto Sánchez-Sánchez, Alejandro Cabrera-Wrooman, Maria Margarita Canales-Martinez, Marco Aurelio Rodriguez-Monroy

**Affiliations:** 1 Laboratorio de Investigación Biomédica en Productos Naturales, Carrera de Medicina, UNAM, FES-Iztacala, Avenida de los Barrios Número 1, Tlalnepantla 54090, Estado de México, Mexico; daniebalcord@gmail.com (D.B.-C.); octaviocanalesa@gmail.com (O.C.-A.); 2 Laboratorio de Génetica Toxicológica, Instituto Politécnico Nacional, Escuela Nacional de Ciencias Biológicas, Av. Wilfrido Massieu, Ciudad de México 07738, Mexico; 3 Unidad de Ingeniería de Tejidos, Terapia Celular y Medicina Regenerativa, Instituto Nacional de Rehabilitación “Luis Guillermo Ibarra Ibarra”, Ciudad de México 14389, Mexico; robsanchez@inr.gob.mx; 4 Laboratorio de Tejido Conjuntivo, Instituto Nacional de Rehabilitación “Luis Guillermo Ibarra Ibarra”, Ciudad de México 14389, Mexico; acabrera@inr.gob.mx; 5 Laboratorio de Farmacognosia, UBIPRO, UNAM, FES-Iztacala, Avenida de los Barrios Número 1, Colonia Los Reyes Iztacala, Tlalnepantla 54090, Estado de México, Mexico; dra.margaritacanales@gmail.com

**Keywords:** propolis, bee products, wound healing, skin histology, anti-inflammatory

## Abstract

Skin wound healing is a complex biochemical process of tissue repair and remodeling in response to injury. Currently, the drugs used to improve the healing process are inaccessible to the population, are costly, and have side effects, making the search for new treatment alternatives necessary. Propolis is a natural product produced by bees that is widely recognized and used in folk medicine for its multiple biomedical activities. However, therapeutic information regarding Mexican propolis is limited. This study aimed to evaluate the wound-healing effect of the Chihuahua ethanolic extract of propolis (ChEEP). Macroscopic and histological analyses were performed using a mouse wound-healing model. The topic acute toxicity assay showed that propolis at 10% w/v had no toxic effects. ChEEP has antibacterial activity against the Gram-positive bacteria *Staphylococcus aureus* and *Staphylococcus epidermidis*. Moreover, it exhibited good anti-inflammatory activity evaluated through mouse ear edema induced by 12-O-tetradeca-noylphorbol-13-acetate (TPA). A full-thickness incision lesion was created in mice and treated topically with 10% ChEEP. At Day 14 post-treatment, it was observed that propolis increased wound contraction and reduced healing time and wound length; furthermore, propolis increased the tensile strength of the wound, as determined with the tensiometric method, and promoted the formation of type I collagen at the site of injury, as evaluated with Herovici stain. These findings suggest that the topical administration of ChEEP can improve skin wound healing, probably due to the synergistic effect of its components, mainly polyphenols, in different steps of the wound-healing process. It should be noted this is the first time that the wound-healing activity of a Mexican propolis has been evaluated.

## 1. Introduction

The skin, the largest organ of the body, is a fibroelastic membrane that plays a crucial role in different processes, such as hydration, thermal regulation, immune recognition, reception of sensory stimuli and vitamin D synthesis initialization. It also acts as a physical barrier, providing protection from ultraviolet radiation, chemicals, and pathogens [1,2,3]. Frequent exposure to external aggressions makes this organ susceptible to injuries that compromise its integrity, altering the normal development of its functions [4]. As a consequence of injury, the wound-healing mechanism, a complex and multifactorial biological process responsible for regaining tissue integrity and restoring local homeostasis, is activated [2,5]. The physiological process consists of a series of cascading yet overlapping stages that can be divided into three main phases: inflammatory, proliferative and remodeling. These processes require highly coordinated and precise signaling from various cells, such as keratinocytes, fibroblasts and endothelial cells that produce an array of cytokines, growth factors and collagen [6,7]. During these stages, different events occur, such as inflammation, cellular phase (granulation), narrowing of wound area (wound contraction), collagen deposition (collagen formation), epithelial covering (epithelization), and scar remodeling (cicatrization). The smooth progression of all these events leads to successful wound healing [2].

Self-regeneration of the skin is generally a considerably fast and efficient process. However, extensive injuries, deep burns, and some diseases like diabetes can compromise normal skin healing [8]. These events could cause alterations that disrupt the healing process and would extend tissue damage, favor an infection, and prolong the repair process, resulting in pathological scenarios such as chronic wounds, diabetic ulcers, psoriasis and pressure ulcers [2,3,7]. 

Skin lesions are a serious public health problem that represents several challenges both for the individuals who develop them (causing pain, discomfort and expenses) and for the world’s health systems, generating substantial morbidity and considerable health care costs. It is estimated that roughly 8.2 million people suffer from wounds with or without infections, with medication costs ranging from USD 28.1 billion to USD 96.8 billion worldwide [9,10]. In Mexico, traumatic wounds (27.5%) and diabetic foot ulcers (24.4%) represent the highest demand for care, with direct monthly costs estimated at MXN 46,563,070.76 (outpatient) and MXN $1,864,124,436.89 (hospitalization) [10].

Although there have been advances in the treatment of wounds, the most effective clinical treatment remains a challenge [11]. Skin damage is often accompanied by inflammation and bacterial infections. Therefore, an ideal therapy should not only promote rapid healing that requires fast wound contraction, shorter epithelization period and adequate gain of tensile strength but also acts as an anti-inflammatory, antibacterial and anti-scarring therapy. Many of the drugs currently used for the treatment of wounds have single pharmacological activity, are expensive, generate side effects such as allergy and drug resistance and are not available in underserved areas [1,7,12]. For these reasons, the search for natural compounds to stimulate tissue repair has gained importance in recent years [13,14].

A variety of natural products or their derivatives have been considered potential candidates for wound healing due to their medicinal properties. These activities are related to their bioactive phytochemical constituents, which could modulate more than one phase of the wound-healing process [2,7,14,15]. Furthermore, factors such as national biodiversity and lower cost make natural products and their derivatives important and accessible therapeutic alternatives for the population [16].

A natural product widely known and used since antiquity in folk medicine to treat different diseases and skin problems is propolis [17,18]. Propolis is a resinous substance produced by bees by adding wax and salivary enzymes to buds and exudates collected from different botanical sources. Bees use it to coat the inner walls, repair cracks in the hive, shelter against rain and wind, embalm intruders and defend against microorganisms [18,19].

The chemical composition of propolis is quite diversified and depends on the botanical origin, time of collection, and geographical and environmental conditions [20]. To date, more than 300 constituents have been identified in propolis samples from around the world, including flavonoids, cinnamic acid derivates, benzoic acids, amino acids, phenolic acids, phenolic aldehydes, terpenes, chalcones, aromatic acids, esters, fatty acids and inorganic compounds [7,18]. This variation in composition, and therefore in the presence and concentration of secondary metabolites, confer several biomedical activities to propolis, most of which are attributed to flavonoids [7,17,18,21].

Propolis extracts from different parts of the world have been reported to have antioxidant, antibacterial, antifungal, anti-inflammatory, antiseptic, anesthetic, photoprotective, immunomodulatory and antitumor activities [7,18,20,22]. They have also shown beneficial effects on skin problems such as psoriasis, ulcers, acne, thrush and in the wound-healing process, especially in the treatment of infectious wounds, acute wounds, burns, and scalds [20,23,24]. However, compared with propolis from other latitudes, such as Brazil, there are few studies on the biomedical properties of Mexican propolis.

Given that wound healing is a complex event, in recent years, efforts have been made to discover a pro-healing agent that shortens the process, decreases cost, is easily available and has minimal side effects on the patient [7]. In this regard, propolis has been shown to be a promising alternative to promote tissue repair and has ideal therapeutic effects [24,25].

Globally research has been dedicated to studying the biomedical properties and chemical composition of propolis from various geographical and climatic regions. Mexico, being a megadiverse country, has numerous amounts of propolis that differ in their chemical composition; however, there are few studies about it [26]. Recently, high antioxidant activity and elevated phenolic and flavonoid contents from Chihuahua propolis (northern Mexico) have been reported [27,28]. Hence, the present work aimed to evaluate the wound-healing potential of the Chihuahua ethanolic extract of propolis. It should be noted this is the first time that the wound-healing activity of a Mexican propolis has been evaluated.

## 2. Results

In a previous study carried out by the work team, the chemical composition of the ChEEP was analyzed by High-Performance Liquid Chromatography–Diode Array Detection (HPLC-DAD) and Liquid Chromatography–Mass Spectrometry (HPLC-MS) [27]. Eight Compounds were identified according to their absorption maxima under low ultraviolet light (λmax) and their retention times for HPLC-DAD; the same compounds were identified for HPLC-MS according to the retention times and parent ion (Table 1).

### 2.1. Acute Dermal Toxicity of ChEEP

**Figure 1:**
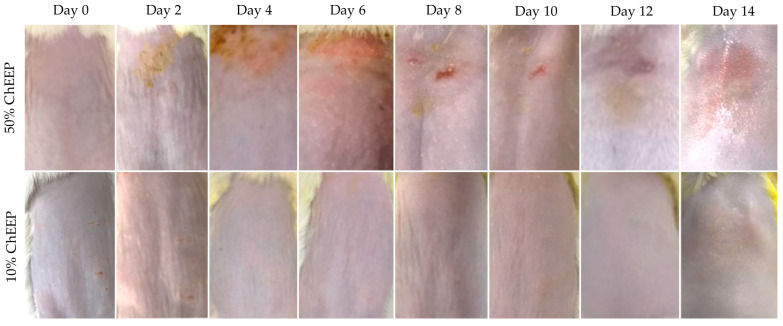
Acute dermal toxicity of ChEEP. Photographs of the dorsal zone skin of the mice where treatments were applied. The upper part was treated with 50% ChEEP, and the lower part was treated with 10% ChEEP ointment.

**Figure 2:**
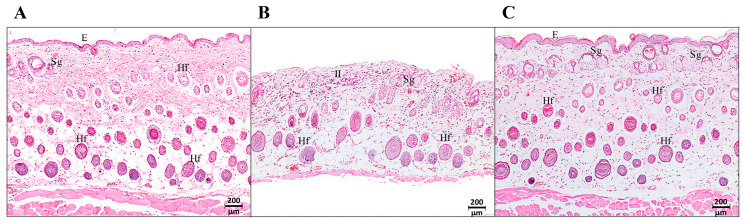
Histology of the acute dermal toxicity test of ChEEP. Photomicrographs of mouse skin 14 days post-treatment in the three experimental groups: untreated (healthy skin) (**A**), with 50% (**B**) and 10% (**C**) ChEEP formulation. Epidermis (E), inflammatory infiltrate (II), hair follicles (Hf) and sebaceous glands (Sg) are indicated. Hematoxylin-eosin staining is shown.

To evaluate if the extract generated any sign of adverse skin reaction in the experimental animals, an acute dermal toxicity assay was carried out according to the Organization for Economic Cooperation and Development (OECD) protocol [29]. Mice were treated topically with 10 or 50% of ChEEP. The ones treated with 50% ChEEP, the highest concentration, showed erythema and edema on the fourth day post-treatment; on day eight, excoriation and ulceration; on the 12th, the appearance of hair; and on the 14th, a slight shine was observed in the area where the extract was applied (Figure 1, upper part). Histological analysis of the skin on the 14th day revealed the presence of abundant inflammatory infiltrate, loss of the normal architecture of the skin, decreased thickness of the skin and muscle, and a greater number of blood vessels and sebaceous glands compared to that of the control group (Figure 2). The almost total loss of the upper layer of the epidermis (stratum corneum) was even observable, which is consistent with the shiny appearance observed at the macroscopic level. Otherwise, the mice treated with 10% ChEEP did not show any signs of irritation, inflammation, ulceration or change in skin coloration at the macroscopic level (Figure 1, lower part); from day eight, the appearance of hair was observed. The microscopic level showed a complete and well-organized skin architecture where the three main layers were distinguished (epidermis, dermis, and hypodermis), and there were an abundance of sebaceous glands and hair follicles in different maturing stages (Figure 2C). These characteristics were similar to those of healthy skin in the control group (Figure 2A). Notably, no change in behavior or mortality was observed in any experimental group during the 14 days that the experiment lasted. Since ChEEP at 10% did not have a toxic effect on the skin of the experimental organisms, it was decided to use this concentration in the following tests.

### 2.2. Antibacterial Activity of ChEEP

**Figure 3:**
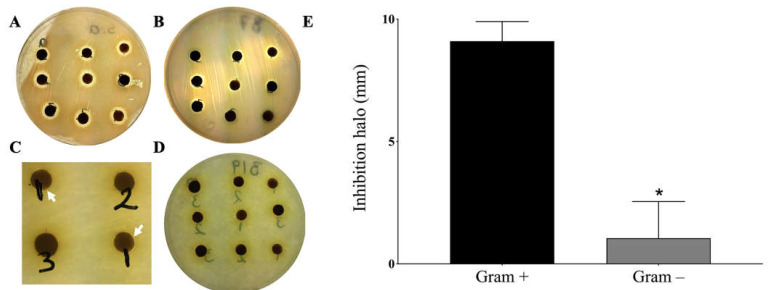
Antibacterial activity of ChEEP. Inhibition halos of Gram-positive bacteria *S. aureus* (**A**) and *S. epidermidis* (**B**) and Gram-negative bacteria *P. aeruginosa* (**C**) and *E. coli* (**D**). White arrowheads indicate small inhibition halos. (**E**) Comparison of the effect of ChEEP between Gram-positive and Gram-negative bacteria. Significant differences were determined by an unpaired “*t*” test. * Indicates significant differences from Gram-positive bacteria.

Since wounds are often invaded by microorganisms, mainly bacteria, a trial was conducted to find out the effect of ChEEP on the most common bacteria present in wounds. Propolis showed to inhibit the growth of the two Gram-positive bacteria *Staphylococcus aureus* and *Staphylococcus epidermidis* at the three concentrations tested, 6, 12 or 18 mg of ChEEP (Figure 3A,B); however, there were no statistically significant differences between them. The results contrasted with Gram-negative bacteria, where minimal inhibition of *Pseudomonas aeruginosa* was found at the lowest concentration tested (6 mg), whereas with *Escherichia coli*, no trace of inhibition was observed at any of the concentrations tested (Figure 3C,D, respectively). The statistical analysis showed that there was a significant difference between the inhibition halos of Gram-positive and Gram-negative bacteria (Figure 3E). It is worth mentioning that all the strains were sensitive to chloramphenicol. 

### 2.3. Topical Anti-Inflammatory Activity of ChEEP

**Figure 4:**
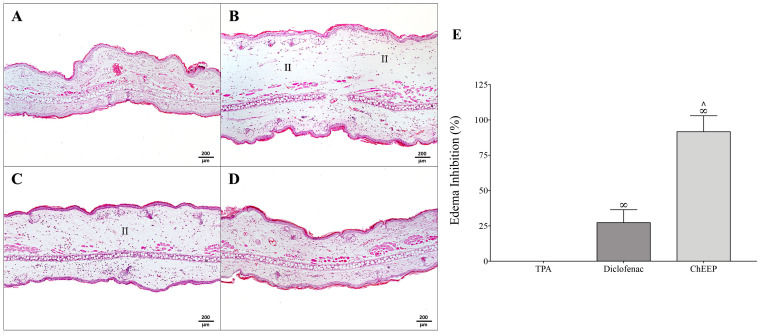
Anti-inflammatory activity of ChEEP. Photomicrographs of mouse ears from the TPA-induced test groups are shown as control (without treatment) (**A**), TPA (**B**), TPA + diclofenac (**C**), and TPA + ChEEP **(D**). Percentage of edema inhibition compared with the control group (left ear-untreated) (**E**). Significant differences were determined by ANOVA. ∞ indicates significant differences with TPA and ∧ with diclofenac. Inflammatory infiltrate (II). Hematoxylin-eosin staining.

Inflammation has an important role in the tissue repair process—it is a natural and necessary process for healing. However, persistent inflammation is directly linked with the delay in wound healing and wound chronicity [5]. In order to evaluate the anti-inflammatory effect of ChEEP, the ear edema test was induced by TPA. The left ears of the mice were the controls, which did not undergo any procedure and therefore had normal histological characteristics (Figure 4A). The positive control group (TPA) showed the presence of inflammatory infiltrate and significantly increased thickness of the ear (Figure 4B), as expected since this compound boosts vasodilation and vascular permeability, causing inflammation. The TPA plus diclofenac group also exhibited inflammatory infiltrate, although in a smaller quantity than in the positive group, and a slight decrease in the thickness of the ear was observed compared with the TPA group (27.3%) (Figure 4C). In the group treated with 10% ChEEP, practically no inflammatory infiltrate was observed, and the histological architecture and ear thickness were very similar to those of the control (Figure 4D). Propolis reduced the inflammation caused by TPA, decreasing ear thickness by 91.6%, which was significantly different from the TPA and diclofenac groups (Figure 4E).

### 2.4. Wound-Healing Efficacy of ChEEP

**Figure 5:**
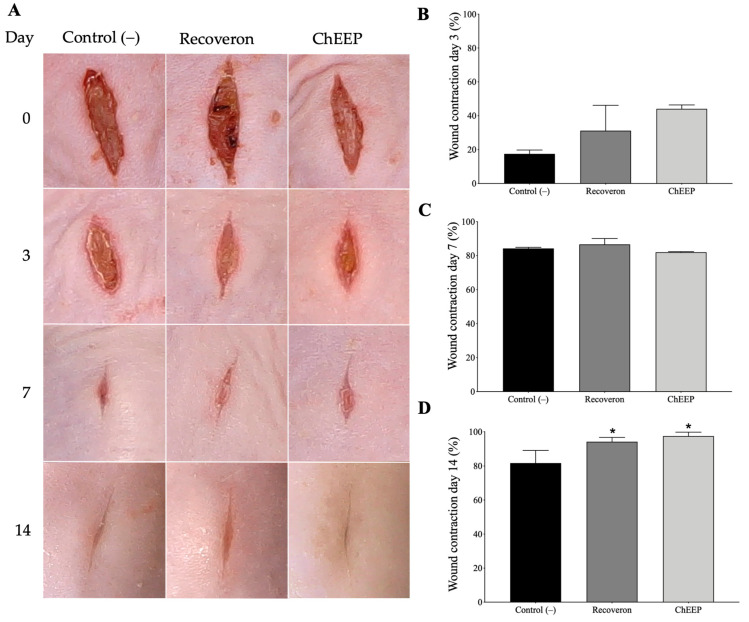
Effect of 10% ChEEP on wound contraction. (**A**) Photographs of the incision wound of the three experimental groups evaluated four times (Day 0- no treatment was applied yet). Percentage of wound contraction evaluated three times, on Day 3 (**B**), 7 (**C**) and 14 (**D**) post-treatment. Significant differences were determined by ANOVA. * Indicates significant differences with respect to the negative control.

To evaluate the influence of ChEEP on the tissue repair process, macroscopic and histological analyses were performed at different experimental times (Days 3, 7 and 14) in a wound incision model. For the wound model, 1 cm long incisions were made in the dorsal skin area of the mice. The wounds of the negative (untreated) and positive (recoveron-treated) control, as well as those of the experimental (ChEEP-treated) group, looked very similar on Days 3 and 7, but on Day 14, the ChEEP-treated wounds appeared more closed compared to the other groups (Figure 5A). Statistical analysis showed that the recoveron and 10% ChEEP groups did not differ from the control on Days 3 and 7; however, both treatments significantly increased wound contraction on Day 14 (Figure 5B–D).

#### 2.4.1. Histology at Day 3 Post-treatment

**Figure 6:**
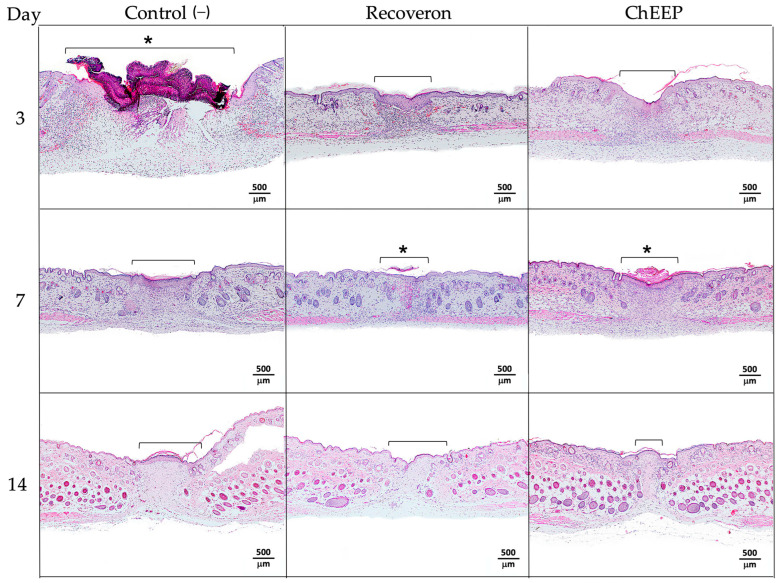
Microscopic effect of 10% ChEEP on wound contraction. Experimental group skin photomicrographs, negative control (1st column), recoveron (2nd column) and ChEEP group (third column), on Days 3, 7 and 14 post-treatment. The wound zone characterized by the loss of architecture and skin structure was indicated with a bracket. * Shows the presence of scar. Hematoxylin-eosin staining.

On the third day post-treatment, a large area with loss of architecture and skin structures was observed in the control group, indicating the area of the wound; likewise, it showed abundant inflammatory infiltrate, a large scar on the lesion and thickening of the epidermis in the areas closest to the wound. In the group treated with recoveron and ChEEP, a lower inflammatory infiltrate and less thickening of the epidermis were observed, as well as a lesser extent of the wound compared to the control group and reepithelization in the damaged area (Figure 6, first row).

#### 2.4.2. Histology at Day 7 Post-treatment

On the seventh day, a decrease in inflammatory infiltrate was observed in the control group compared to day three; the presence of hair follicles and reepithelization in the wound area was found, although thickening of the epidermis remained. In the groups treated with recoveron and ChEEP, slight and moderate inflammatory infiltrates were observed, respectively. In both groups, the epidermis decreased in thickness, and the presence of hair follicles was detected (Figure 6, second row).

#### 2.4.3. Histology at Day 14 Post-treatment

On Day 14, slight inflammatory infiltrate and more hair follicles were found, while thickening of the epidermis persisted in the control group. In contrast, in the recoveron group, the thickness of the epidermis decreased, the inflammatory infiltrate was minimized, and a greater number of hair follicles was observed. The ChEEP group showed an evident shortening of the wound area, a reduction in the amount of inflammatory infiltrate and more hair follicles in different stages of maturation, which were visibly well organized compared with those of the other groups (Figure 6, third row).

The experimental group treated with surgical gel, the vehicle in which ChEEP was diluted and applied, did not show macroscopic and histological differences in the healing process with respect to the control group during the 14 days of the experiment. 

#### 2.4.4. Wound Length

**Figure 7:**
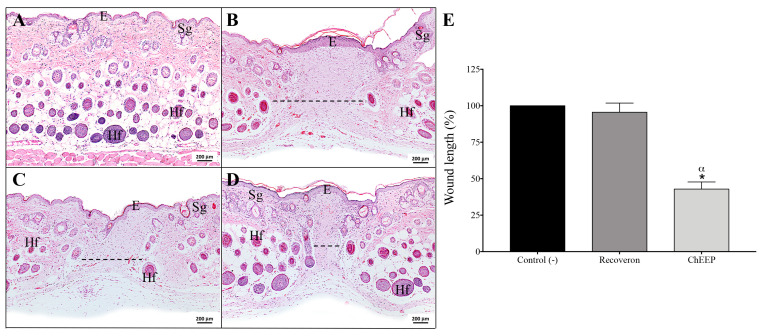
Effect of 10% ChEEP on wound length. Skin photomicrographs of each group were observed: healthy skin (**A**), negative control (**B**), recoveron (**C**) and ChEEP group (**D**). The wound is indicated with dotted lines over the images. Percentage of wound length in the wound contraction test samples on Day 14 (**E**). Significant differences were determined by ANOVA. * Indicates significant differences with respect to the negative control. ^α^ Indicates significant differences with respect to the recoveron group. Epidermis (E), hair follicles (Hf) and sebaceous glands (Sg) are marked. Hematoxylin-eosin staining is shown.

In order to assess wound length, the lesion area of each photomicrography was measured end-to-end with ImageJ software (v. 1.52). Graphing these data confirmed that ChEEP significantly reduced the length of the wound compared with the negative control and recoveron groups on Day 14 (Figure 7).

#### 2.4.5. Tensile Strength

**Figure 8:**
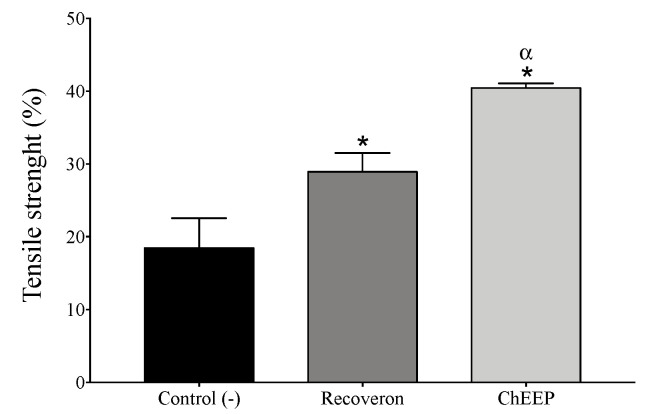
Tensile strength of ChEEP. Percentage of tensile strength in the experimental groups compared with that of the reference group (healthy skin). Significant differences were determined by ANOVA. * Indicates significant differences with respect to the negative control. ^α^ Indicates significant differences from the recoveron treatment group.

To evaluate the strength with which the wound closes, the tensiometric method was used. The mean tensile strength in the negative control and recoveron groups tended to increase by approximately 18.4% and 28.9%, respectively, compared to that of the reference group (healthy skin), while that of the ChEEP group increased further by 40.4%. All groups presented significant differences in mean tensile strength (Figure 8).

#### 2.4.6. Effect of ChEEP on Collagen Formation

**Figure 9:**
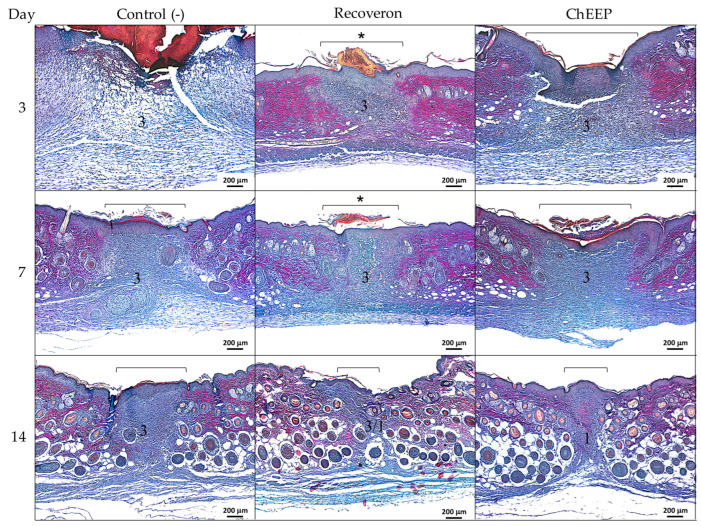
Effect of 10% ChEEP on collagen formation. Experimental group skin photomicrographs, negative control (1st column), recoveron (2nd column) and ChEEP group (3rd column), on Days 3, 7 and 14 post-treatment. Collagen type 1 stained in magenta (1) and type 3 in blue (3). The wound zone characterized by the loss of architecture and skin structure was indicated with a bracket. * Shows the presence of a scar. Herovici stain was used.

The last stage of wound healing is the remodelling phase, in which type III collagen fibers are gradually replaced by type 1, and the tensile strength of the newly formed tissue increases by changing the composition of the extracellular matrix (ECM) [11]. Due to that, ChEEP was shown to increase the tensile strength, and the type of collagen present in the wound was evaluated by Herovici staining, which discriminates between collagen I (magenta) and III (blue). Histological analyses revealed that the three experimental groups (negative control, recoveron and ChEEP) showed a blue coloration on the wound area on Day 3, which indicated the presence of type III collagen (Figure 9, first row). The same color pattern was observed in all groups on Day 7 (Figure 9, second row). However, on Day 14, in the negative control group, blue coloration continued. In the recoveron group, a mixture of blue and magenta was observed, while in the ChEEP group, magenta predominated, indicating the presence of type I collagen (Figure 9, third row). The results revealed that ChEEP favors the formation of collagen type I on Day 14 compared to the other experimental groups.

## 3. Discussion

Interest in alternative medicines and natural products for the treatment of skin has increased in recent years due to the lower incidence of side effects, as well as better economic and physical accessibility than currently available medicines. Propolis is a natural product processed by bees from different plant species; it has great variability in chemical composition, which gives it multiple biomedical activities.

Propolis is normally well tolerated, with rare incidents of allergic reactions and toxicity [18]. It was observed that ChEEP applied topically at 10% concentration does not produce alterations at the macro and microscopic levels, which agrees with that reported for Brazilian green propolis (30%) [30]. Otherwise, 50% of ChEEP showed signs of mild irritation. In the histological analysis, abundant inflammatory infiltrate, decreased skin thickness and loss of the outermost layer of the epidermis (stratum corneum) were found. Based on these results, 50% ChEEP is considered to be a slight irritant [31]. It has been described that high concentrations of propolis have antiproliferative and cytotoxic activities in skin cell cultures [6,32], which could explain the decreased skin thickness and loss of the outermost layer of the epidermis.

Wounds provide a moist, warm and nourishing environment that promotes microbial colonization, growth and infection, mostly in the case of chronic wounds [33,34]. Although bacteria are a common part of the intact skin microbiota and wounds, a critical threshold of existing bacteria and the formation of a biofilm may impede wound healing. Therefore, control of infection is a crucial step in the healing process [34,35,36]. *S. aureus*, followed by *P. aeruginosa* and *E. coli*, are the most prevalent organism associated with wound infection, although polymicrobial infection is also usual. These strains have been identified as multidrug-resistance, which complicates their treatment [33,37]. In the present study, ChEEP showed antibacterial activity; however, this effect was better against Gram-positive than Gram-negative bacteria. The same behavior has already been reported with propolis from Brazil, Canada, Africa and Mexico (Sonora) [38,39,40,41,42]. The antibacterial activity of propolis has been attributed to the synergism of the phenolic acids and flavonoids in its chemical composition; these molecules inhibit the nucleic acid synthesis and depolarize the cytoplasmic membrane, which leads to bacterial death [18,40,43,44]. Accordingly, ChEEP was reported to have a high proportion of phenolic compounds [27]. However, the variation in the effect between both types of bacteria may be due to the structural differences in the cell wall; Gram-negative bacteria have a more chemically complex wall, with the presence of the negatively charged lipopolysaccharides, which act as a barrier, providing greater resistance [45].

On the other hand, the inflammatory response is a highly synchronized natural process, crucial and necessary for the wound-healing process. It allows the wound bed to be healed by removing necrotic tissue, debris, and bacterial contaminants, as well as recruiting and activating fibroblasts. However, an exacerbated inflammatory state may result in deregulated phases of the healing process, extending tissue damage, oxidative stress and repair time, which can trigger a chronic wound or may lead to excessive scarring [2,12,46]. In the inflammatory assay induced by TPA, ChEEP showed an edema inhibition of approximately 91%, which is considered a good anti-inflammatory effect (edema inhibition > 65%) [47]. The topical administration of TPA triggers acute edema with leucocyte infiltration through the activation of protein kinase C (PKC). PKC, in turn, activates Nuclear factor kappa B (NF-κB), promoting the expression of several proinflammatory agents, such as cyclooxygenase 2 (COX-2), inducible nitric oxide synthase (iNOS) and inflammatory cytokines, such as Interleukin-1 (IL-1), IL-2, IL-6, IL-8 and Tumor necrosis factor alpha (TNF-α) [48,49]. Previously, an extract of propolis from Chiapas (Mexico) and its major isolated compound pinocembrin (previously reported in ChEEP composition), showed anti-inflammatory activity in a TPA model by decreasing the levels of myeloperoxidase, an enzyme produced by neutrophils that increases its activity when an inflammatory process is triggered by TPA administration [50]. In this regard, propolis has been reported to exert anti-inflammatory activity, decreasing the generation of prostaglandins and leukotrienes due to inhibition of the expression and activities of COX and lipoxygenases, delaying the expression of iNOS, inhibiting TNF-α mediated by NF-κB and reducing the immune response in T cells [49,51]. Moreover, some propolis has been shown to decrease the release of nitric oxide (NO), an important inflammatory mediator generated by iNOS. The main inhibitory components of NO release have been determined to be chrysin, kaempferol, quercetin and galangin [52], and the first three compounds were identified in ChEEP by HPLC [27]. In addition, Brazilian propolis has been shown to inhibit the chemotaxis of human polymorphonuclear leukocytes (PMNs), which are related to the exacerbation of inflammatory reactions [53]. Therefore, ChEEP could produce an anti-inflammatory effect against TPA through NF-κB, iNOS and PMNs chemotaxis inhibition. 

Wound healing involves many mechanisms, such as hemostasis, inflammation, synthesis and deposition of matrix, angiogenesis, fibroplasia, reepithelization, contraction, and remodeling. Any alteration in these events will prolong tissue damage and delay the repair process. From this perspective, various propolis have demonstrated their ability to accelerate wound healing due to their action at different stages of the process [2,7,24]. Propolis has been shown to reduce scar formation, shorten healing time, increase wound contraction, enhance epithelialization, and accelerate tissue repair [24,36,54]. It has also been reported that propolis modulates the distribution of healing-associated factors, mainly collagen I, collagen III and matrix metallopeptidase 9 (MMP-9), involved in the ECM constitution; and the Vascular endothelial growth factor (VEGF), which stimulates angiogenesis during the proliferative phase [54]. It has been described that propolis decreases wound size and inflammation. In addition, on Day 7 post-treatment, propolis increased reepithelization, while on Day 14, it had high fibroblast activity, and the wound area was completely repaired in incisional and burn wound models [55,56], which is consistent with the ChEEP results.

Recently, a systematic review and meta-analysis of more than 40 published studies found strong evidence that propolis is effective in the treatment of skin wounds, including difficult-to-heal ulcers found in patients with diabetes; in fact, propolis was shown to promote a higher percentage of healing than the interventions classically employed [25]. Another interesting aspect is that a large number of in vivo studies have combined propolis with other substances, such as honey, and different biomaterials (nanoparticles, nanofibrous, biocelullose membrane, hydrogel) to achieve additive or synergistic effects in the skin regeneration process [25,57,58,59,60,61,62].

Propolis has also been suggested to stimulate the production of collagen fibers [7,56]. Collagen is a protein molecule synthetized by fibroblasts, which gives structure, strength, and elasticity to the skin. Collagen participates in cell signaling, angiogenesis, the expression of inflammatory cytokines and growth factors, and interactions between matrix metalloproteinases (MMPs) and their tissue inhibitors, which are inherent elements of reepithelization. Collagen types I and III are the main collagen types in healthy skin and are predominantly expressed during the repair process [63]. In the remodeling tissue phase of the wound-healing process, fibroblasts initially produce type III collagen to guide the proliferation and migration of fibroblasts and endothelial cells during granulation tissue formation; then, type III collagen is gradually replaced by type I collagen, which is indispensable for keratinocyte migration and reepithelization, providing tensile strength and mechanical stability to the new connective tissue [20,44,64]. It has been reported that propolis induces a faster replacement of type III to I collagen, better fiber organization, reepithelization of the damaged area and an increase in the amount of myofibroblasts on Day 14, suggesting that propolis enhances fibroblastic activity, as well as collagen synthesis and deposition [63,64,65]. Herovici stain results revealed the presence of type I collagen on wounds treated with ChEEP on Day 14, while the prevalence of type III was still observed in the other groups, suggesting that ChEEP will stimulate and accelerate the replacement of type III to I collagen, explaining the significant increase in the tensile strength of the skin (40.4%). These effects have been attributed to the high content of flavonoids present in propolis [64,65]. By inhibiting MMPs (enzymes that break down collagen), flavonoids could increase the rate and amount of collagen necessary for the formation of a new wound matrix, thereby speeding up the healing process [66]. In fact, some flavonoids, such as luteolin, chrysin and kaempferol, previously reported in ChEEP [27], have already been shown to stimulate collagen synthesis and deposition [66,67].

On the other hand, during the healing process, the production of reactive oxygen species (ROS) increases. These molecules are produced as a consequence of normal physiological aerobic metabolism and play a pivotal role in the normal wound-healing response. The moderate elevation of ROS aids in the defense of wounds against microbial infections and promotes vascularization by activating multiple cellular signaling pathways. However, if the endogenous antioxidant system fails to balance the production and degradation of ROS, the excessive levels of ROS create a condition of oxidative stress, which activates pro-apoptotic proteins, generating toxic effects on cells, causing inflammation and delay in the healing process. Exogenous antioxidants such as those found in plant metabolites are an alternative to reduce oxidative damage, having a beneficial effect on wound healing [32,68,69,70,71,72]. In previous studies carried out in our laboratory, the antioxidant capacity (SA_50_) of ChEEP was determined, and the extract showed an SA_50_ = 15.75 μg/mL [27], in agreement with the antioxidant activity index is considered a very strong antioxidant activity [73]. In addition, 31.4% (314 mg equivalent of gallic acid/g) of the total ChEEP extract are phenolic compounds and 6.2% (6.25 mg equivalent of quercetin/g) are flavonoids [27]; both results exceed the minimum parameters established by the Official Mexican standard of production and processing of propolis (NOM-003-SAG/GAN-2017), which establishes a minimum of 5% and 0.5%, respectively [74]. 

Phenolic compounds, mainly flavonoids, have generated great interest in wound treatment due to their anti-inflammatory, angiogenesis, re-epithelization, and antioxidant properties [75,76]. Flavonoids such as pinocembrin, kaempferol, chrysin, naringin, naringenin, quercetin, acacetin, and luteolin were identified in ChEEP by HPLC-DAD and HPLC-MS [27]. These compounds have already been reported to have anti-inflammatory, antioxidant, and antibacterial activities [20,77,78,79,80,81,82,83,84,85,86,87,88], with the exception of naringenin and acacetin, which, compared to the other flavonoids, have been shown to have low antibacterial and antioxidant activities, respectively [87,88]. As described above, skin wound healing involves several sequential steps in which these biomedical properties are crucial to restore skin integrity. In fact, our team has recently attributed the hypoglycemic and gastroprotective effects found with Chihuahua propolis to its good anti-inflammatory and antioxidant activities [27,28].

Previous studies have already reported that flavonoids can act in all phases of wound healing and activate the intracellular signaling pathways essential for healing to occur. They were shown to be able to act on macrophages, fibroblast and endothelial cells by mediating the release and expression of Transforming growth factor β1 (TGF-β1), VEGF, and IL-10. Moreover, they were able to reduce the release of inflammatory cytokines, NFκB and ROS [75,76]. Actually, the flavonoid kaempferol, identified in ChEEP, has already been shown to be an effective topical wound-healing agent in the treatment of non-diabetic and diabetic wounds in a rat model [89]. This background supports the results found in the present study.

An important fact to consider is that Mexico is among the leading producers of honey, ranking sixth in the world. However, there are no reliable statistics available to indicate the production of propolis [90]. Beekeepers are unaware of the potential of certain bee products, such as propolis, and frequently discard them. This situation demonstrates the importance of scientific validation of this product and the urgency to educate beekeepers and society on these findings so that they can obtain their benefits.

When extrapolating findings from a mouse model to the human population, it is crucial to consider physiological differences between mice and humans. It highlights variations in skin physiology, immune response, and pharmacokinetics as crucial factors to consider.

The structure and function of the skin can differ between species, with mice having a thinner epidermis and higher hair follicle density than humans, potentially impacting the penetration and absorption of topical treatments. Variances in the presence and distribution of skin components, such as sebaceous and sweat glands, may also affect the efficacy and safety of propolis.

Additionally, the immune response and inflammatory processes involved in wound healing can vary between mice and humans. Mice may exhibit a more robust or different immune response compared to humans, meaning that the effects of propolis on immune modulation and inflammation observed in mice may not directly translate to humans [91,92].

Furthermore, substances’ absorption, distribution, metabolism, and excretion can vary across species. Differences in metabolism, bioavailability, and clearance rates between mice and humans can influence the concentration and duration of propolis components in the skin. Considering these pharmacokinetic differences is crucial when extrapolating dosages used in animal studies to potential clinical applications [93].

Given these factors, caution should be exercised when applying results from animal studies, such as the mouse model used in this research, to clinical settings. Further studies, including well-controlled clinical trials, are necessary to evaluate the safety and efficacy of propolis in humans, determine appropriate dosage regimens, and identify potential side effects.

Therefore, while findings from the mouse model provide valuable insights and serve as a foundation for future research, it is essential to recognize the limitations and bridge the gap between preclinical and clinical studies to assess the potential of propolis in human skin conditions fully.

## 4. Materials and Methods

### 4.1. Propolis

Mexican propolis samples were collected by Ing. Martín Balcorta Baeza in 2013 from the apiary located in Ejido Concordia, in the municipality of Aquiles Serdán and Chihuahua (Chihuahua, Mexico). ChEEP was obtained by the maceration method described previously [27]; in this process, ethanol was used as a solvent. Once the dissolved extract was obtained, it was taken to a solvent evaporation process through distillation at reduced pressure in a rotary evaporator. The extract was free of the solvent. Ethanolic extract was selected because it is the way available for the population.

### 4.2. Ointment Preparation with ChEEP 

UltraSonic conductive gel was used as a vehicle to apply the ChEEP. The propolis ointment was prepared in a ratio of 10% and 50% *w*/*v*. Ten milliliters of the formulation was prepared for each assay.

### 4.3. Experimental Animals

Seven-week-old male genetic origin CD-1 mice (*Mus musculus*) were used. Animals were housed in a well-ventilated room at 25 °C under a light-dark cycle in polycarbonate cages with food and water *ad libitum*. The present study protocols were approved by the Institutional Ethics Committee of the Universidad Nacional Autónoma de México (UNAM), Facultad de Estudios Superiores Iztacala (FESI), according to the guidelines of the Federal Regulations for Animal Experimentation and Care (NOM-062-ZOO-1999, Ministry of Agriculture, Mexico City). The experimental animals were randomly assigned in groups of five animals each (*n* = 5) and received treatments according to the objective of each trial.

### 4.4. Acute Dermal Toxicity

Animals showing normal skin texture were acclimatized to the laboratory conditions for five days prior to the test. Twenty-four hours before the study, we shaved the dorsal area of the trunk of the test animals, approximately 10% of the body surface area. The trial was conducted in accordance with OECD guidelines [29]. The experimental animals were randomly assigned into three groups: one control group, without treatment; two experimental groups, treated with 10% and 50% ChEEP, which received two daily doses of 50 mg over the shaved area for 14 days. The animals were observed for the development of any adverse skin reaction and behavior patterns, and photos of the area were taken daily for 14 days. At the end of the exposure period, residual test substance was removed, and a skin sample was obtained for histological analyses (modified from [12]).

### 4.5. Antibacterial Activity of CHEEP

The Gram-positive bacteria *Staphylococcus aureus* and *Staphylococcus epidermidis*, in addition to the Gram-negative bacteria *Pseudomonas aeruginosa* and *Escherichia coli*, were donated by the Laboratory of Pharmacognosy of FESI, UNAM. The antibacterial activity was evaluated using the Kirby-Baüer method [94]. Five-millimeter diameter disks were coated with 6, 12 or 18 mg of ChEEP, disks with 25 μg chloramphenicol (Sigma-Aldrich, Saint Louis, MO, USA) were used as a positive control, and the tests were performed in triplicate. After 24 h, the inhibition halo around the disks was measured.

### 4.6. Topical Anti-Inflammatory Activity of ChEEP

The ear edema test model induced by 12-O-tetradeca-noylphorbol-13-acetate (TPA) (Sigma-Aldrich, Saint Louis, MO, USA) was used [48]. TPA was dissolved in ethanol (2.5 µg/20 mL), and the solvent did not interfere with the assay. The experimental animals were randomly assigned into three groups: TPA (only positive control), TPA plus diclofenac (Novartis, Mexico City) (nonsteroidal anti-inflammatory drug, negative control) and TPA plus ChEEP. The left ears of the mice were not treated and were the control in each organism. The animals were fasted for 4 h prior to the experiment. Subsequently, they were anesthetized with 250 µL of isoflurane (PISA, Mexico City, Mexico) (inhalation anesthetic), and 10 µL of TPA was administered topically to the inner and outer surface of the right ear of each group. Thirty minutes after the application of TPA, animals received 10 µL of diclofenac or 10 mg of ChEEP on both sides of the right ear. Mice were sacrificed by cervical dislocation after 4 h, and a circular punch biopsy (5 mm) of the right and left ears was taken and fixed in formalin. The histological evaluation included H&E staining and ear thickness was measured with the ImageJ program. Inflammation was assessed by the increase in thickness of the right ear compared to that of the left ear of each of the experimental organisms.

### 4.7. Wound-Healing Efficacy of ChEEP

#### 4.7.1. Experimental Protocol of Wound Incision

Twenty-four hours prior to the trial, the dorsal area of the trunk of the test animals was shaved. Mice were anesthetized with 250 µL of isoflurane, and incisions 1 cm long were made through the skin and subcutaneous tissue. Animals were randomly distributed, and four experimental groups were established: negative control without treatment; positive control treated with recoveron NC^®^ (Armstrong Laboratorios, Mexico City), a commercial wound-healing drug; propolis group treated with 10% ChEEP formulation; and a reference group with healthy skin without any treatment. Every treatment was applied topically over the lesion area twice a day, as described in the acute dermal toxicity assay. The incision area was photographed daily with the digital manual microscope Celestron (Torrance, CA, USA) to determine the wound contraction area with the ImageJ program. The contraction data were expressed as a percentage of the initial lesion area. Mice were sacrificed by cervical dislocation at 3, 7 or 14 days post-treatment, and skin samples from the lesion site were obtained and processed for histological evaluation, which included H&E and Herovici staining. Photomicrographs were taken, and the histological architecture of the wound was compared at the three times evaluated. The results were expressed as the percentage of wound closure based on the size difference between the lesions of the groups.

#### 4.7.2. Tensile Strength Model

The tensiometric method was used to measure the tensile strength of the skin (modified from [12]). Mice were shaved, anesthetized, and incised as described for the wound contraction assay. The same four experimental groups were established for the wound incision model; reference (healthy skin), negative control (without treatment), positive control (recoveron NC^®^) and treatment with the 10% ChEEP group. After 14 days of treatment, the mice were sacrificed, and the tensile strength was taken to be the load in grams required to disrupt the wound. The results were expressed as the percentage of tensile strength.

### 4.8. Statistical Analysis

All data concerning antibacterial and anti-inflammatory activity, as well as wound-healing efficacy (wound closure, length, and tensile strength), were expressed as the means ± SDs. Statistical differences between the treatments and controls were tested by one-way analysis of variance (ANOVA), while an unpaired “*t*” test was used for the antibacterial activity assay using GraphPad Prism software (8.4.0). A difference in the mean values of *p* < 0.05 was considered statistically significant.

## 5. Conclusions

These results constitute the first in vivo study of the effects of Mexican propolis on the wound-healing process. This investigation demonstrated the anti-inflammatory and wound-healing activity of ChEEP, as evidenced by macroscopic and histological analysis. Considering the antioxidant, anti-inflammatory, antimicrobial, and healing properties attributed to propolis from different parts of the world, in addition to the biomedical activities evaluated in the present study, we can infer that propolis acts integrally in the wound-healing process. In turn, propolis can prevent infection, reduce the wound contraction time, avoid exacerbated inflammation, stimulate the replacement of type III to I collagen, and increase tensile strength and the speed with which the wound closes, making ChEEP a promising alternative to improve the healing process. These effects are related to the high polyphenolic composition of ChEEP. Propolis is a mixture of biochemical compounds that act in synergy, influencing different targets and, therefore, different biomedical activities, such as those evaluated in this study. 

To obtain an integral understanding, further research is needed to elucidate the mechanism of action of Mexican propolis in the wound-healing process. Future studies should include immunohistochemical analyses of proliferation and migration, evaluation of the anti-inflammatory and antioxidant effects of ChEEP directly in the wound model, and pharmacokinetic studies. Moreover, evaluations of propolis’s safety, effective doses, and possible long-term adverse effects should be carried out to promote its use in different therapeutic applications, including wound healing. We hope our studies will provide a scientific basis for future basis and translational research.

## Figures and Tables

**Table 1:** HPLC-DAD and HPLC-MS analysis of ChEEP.

Name	Retention Time (min)		λmax (nm)	Parent Ion (*m*/*z*) [M-H]^−^	Relative Error (ppm)
	HPLC-DAD	HPLC-MS			
Naringin Naringenin Kaempferol Quercetin Acacetin Luteolin Pinocembrin Chrysin	5.71 7.88 8.31 8.94 9.40 10.05 14.82 17.58	17.116 24.071 26.892 23.158 32.32 22.51 30.378 31.175	214, 282 290, 325 200, 266, 366 256, 372 210, 268, 324 252, 348 290 268, 314	315.8400 271.0621 285.0412 301.0361 283.0619 285.0774 255.0672 253.0509	2.98 −3.18 −2.58 −2.48 −2.32 −1.98 −3.34 −1.18

Taken from [27].

## Data Availability

Not applicable.

## References

[1] Medellin-Luna M.F., Castaneda-Delgado J.E., Martínez-Balderas V.Y., Cervantes-Villagrana A.R. (2019). Medicinal plant extracts and their use as wound closure inducing agents. J. Med. Food.

[2] Ibrahim N.I., Wong S.K., Mohamed I.N., Mohamed N., Chin K.-Y., Ima-Nirwana S., Shuid A.N. (2018). Wound healing properties of selected natural products. Int. J. Environ. Res. Public Health.

[3] Tottoli E.M., Dorati R., Genta I., Chiesa E., Pisani S., Conti B. (2020). Skin wound healing process and new emerging technologies for skin wound care and regeneration. Pharmaceutics.

[4] Guarín-Corredor C., Quiroga-Santamaría P., Landínez-Parra N.S. (2013). Proceso de Cicatrización de heridas de piel, campos endógenos y su relación con las heridas crónicas. Rev. Fac. Med..

[5] Corrêa F.R.S., Schanuel F.S., Moura-Nunes N., Monte-Alto-Costa A., Daleprane J.B. (2017). Brazilian red propolis improves cutaneous wound healing suppressing inflammation-associated transcription factor NFκB. Biomed. Pharmacother..

[6] Jacob A., Parolia A., Pau A., Amalraj F.D. (2015). The effects of Malaysian propolis and Brazilian red propolis on connective tissue fibroblasts in the wound healing process. BMC Complement. Altern. Med..

[7] Iyyam Pillai S., Palsamy P., Subramanian S., Kandaswamy M. (2010). Wound healing properties of Indian propolis studied on excision wound-induced rats. Pharm. Biol..

[8] Pereira R.F., Bartolo P.J. (2016). Traditional therapies for skin wound healing. Adv. Wound Care.

[9] Sen C.K. (2019). Human Wounds and Its Burden: An Updated Compendium of Estimates. Adv. Wound Care.

[10] Vela-Anaya G., Stegensek-Mejía E.M., Leija-Hernández C. (2018). Características epidemiológicas y costos de la atención de las heridas en unidades médicas de la Secretaría de Salud. Rev. Enfermería Inst. Mex. Seguro Soc..

[11] Mirhaj M., Labbaf S., Tavakoli M., Seifalian A.M. (2022). Emerging treatment strategies in wound care. Int. Wound J..

[12] Mulisa E., Asres K., Engidawork E. (2015). Evaluation of wound healing and anti-inflammatory activity of the rhizomes of *Rumex abyssinicus* J.(Polygonaceae) in mice. BMC Complement. Altern. Med..

[13] Abu-Ahmed H., Abdel-Wahed R., El-Kammar M., El-Neweshy M. (2013). Evaluation of the effectiveness of propolis compared with honey on second intention wound healing in the equine. Middle East J. Sci. Res..

[14] Tsala D.E., Amadou D., Habtemariam S. (2013). Natural wound healing and bioactive natural products. Phytopharmacology.

[15] Moeini A., Pedram P., Makvandi P., Malinconico M., d’Ayala G.G. (2020). Wound healing and antimicrobial effect of active secondary metabolites in chitosan-based wound dressings: A review. Carbohydr. Polym..

[16] Grillo L.A.M., Dornelas C.B. (2012). Comparative study of topical green and red propolis in the repair of wounds induced in rats. Rev. Col. Bras. Cir..

[17] Barbosa M.H., Zuffi F.B., Maruxo H.B., Jorge L.L.R. (2009). Therapeutic properties of propolis for treatment of skin lesions. Acta Paul. Enferm..

[18] Martinotti S., Ranzato E. (2015). Propolis: A new frontier for wound healing?. Burn. Trauma.

[19] Bonamigo T., Campos J.F., Alfredo T.M., Balestieri J.B.P., Cardoso C.A.L., Paredes-Gamero E.J., de Picoli Souza K., dos Santos E.L. (2017). Antioxidant, cytotoxic, and toxic activities of propolis from two native bees in Brazil: Scaptotrigona depilis and Melipona quadrifasciata anthidioides. Oxidative Med. Cell. Longev..

[20] Kurek-Górecka A., Górecki M., Rzepecka-Stojko A., Balwierz R., Stojko J. (2020). Bee Products in Dermatology and Skin Care. Molecules.

[21] Abu-Seida A.M. (2015). Effect of propolis on experimental cutaneous wound healing in dogs. Vet. Med. Int..

[22] Silva-Carvalho R., Baltazar F., Almeida-Aguiar C. (2015). Propolis: A complex natural product with a plethora of biological activities that can be explored for drug development. Evid.-Based Complement. Altern. Med..

[23] Oršolić N., Skurić J., Đikić D., Stanić G. (2014). Inhibitory effect of a propolis on di-n-propyl disulfide or n-hexyl salycilate-induced skin irritation, oxidative stress and inflammatory responses in mice. Fitoterapia.

[24] Yang J., Pi A., Yan L., Li J., Nan S., Zhang J., Hao Y. (2022). Research progress on therapeutic effect and mechanism of propolis on wound healing. Evid.-Based Complement. Altern. Med..

[25] Machado Velho J.C., França T.A., Malagutti-Ferreira M.J., Albuquerque E.R., Lívero F.A.d.R., Soares M.R., Soares A.E.E., Ribeiro-Paes J.T. (2023). Use of propolis for skin wound healing: Systematic review and meta-analysis. Arch. Dermatol. Res..

[26] Rivero-Cruz J.F., Granados-Pineda J., Pedraza-Chaverri J., Pérez-Rojas J.M., Kumar-Passari A., Diaz-Ruiz G., Rivero-Cruz B.E. (2020). Phytochemical constituents, antioxidant, cytotoxic, and antimicrobial activities of the ethanolic extract of Mexican brown propolis. Antioxidants.

[27] Rivera-Yañez N., Rodriguez-Canales M., Nieto-Yañez O., Jimenez-Estrada M., Ibarra-Barajas M., Canales-Martinez M., Rodriguez-Monroy M. (2018). Hypoglycaemic and antioxidant effects of propolis of Chihuahua in a model of experimental diabetes. Evid.-Based Complement. Altern. Med..

[28] Ruiz-Hurtado P.A., Garduño-Siciliano L., Dominguez-Verano P., Martinez-Galero E., Canales-Martinez M.M., Rodriguez-Monroy M.A. (2021). Evaluation of the gastroprotective effects of Chihuahua propolis on indomethacin-induced gastric ulcers in mouse. Biomed. Pharmacother..

[29] OECD (2017). Test No. 402: Acute Dermal Toxicity. OECD Guidelines for the Testing of Chemicals, Section 4.

[30] Mazia R.S., de Araújo Pereira R.R., de Francisco L.M.B., Natali M.R.M., Dias Filho B.P., Nakamura C.V., Bruschi M.L., Ueda-Nakamura T. (2016). Formulation and evaluation of a mucoadhesive thermoresponsive system containing Brazilian green propolis for the treatment of lesions caused by herpes simplex type I. J. Pharm. Sci..

[31] Draize J., Woodard G., Calevery H. (1944). Methods for the study of irritation and toxicity of substances apllied topically to the skin and mucous membranes. J. Pharmacol. Exp. Therapeutics.

[32] Tyszka-Czochara M., Paśko P., Reczyński W., Szlósarczyk M., Bystrowska B., Opoka W. (2014). Zinc and propolis reduces cytotoxicity and proliferation in skin fibroblast cell culture: Total polyphenol content and antioxidant capacity of propolis. Biol. Trace Elem. Res..

[33] Alam M.M., Islam M.N., Hawlader M.D.H., Ahmed S., Wahab A., Islam M., Uddin K.R., Hossain A. (2021). Prevalence of multidrug resistance bacterial isolates from infected wound patients in Dhaka, Bangladesh: A cross-sectional study. Int. J. Surg. Open.

[34] Rahim K., Saleha S., Zhu X., Huo L., Basit A., Franco O.L. (2017). Bacterial contribution in chronicity of wounds. Microb. Ecol..

[35] Negut I., Grumezescu V., Grumezescu A.M. (2018). Treatment strategies for infected wounds. Molecules.

[36] Oryan A., Alemzadeh E., Moshiri A. (2018). Potential role of propolis in wound healing: Biological properties and therapeutic activities. Biomed. Pharmacother..

[37] Bessa L.J., Fazii P., Di Giulio M., Cellini L. (2015). Bacterial isolates from infected wounds and their antibiotic susceptibility pattern: Some remarks about wound infection. Int. Wound J..

[38] Berretta A.A., Nascimento A.P., Bueno P.C.P., Leite M.M.d.O.L. (2012). Propolis standardized extract (EPP-AF^®^), an innovative chemically and biologically reproducible pharmaceutical compound for treating wounds. Int. J. Biol. Sci..

[39] Rahman M.M., Richardson A., Sofian-Azirun M. (2010). Antibacterial activity of propolis and honey against Staphylococcus aureus and Escherichia coli. Afr. J. Microbiol. Res..

[40] Nedji N., Loucif-Ayad W. (2014). Antimicrobial activity of Algerian propolis in foodborne pathogens and its quantitative chemical composition. Asian Pac. J. Trop. Dis..

[41] Velazquez C., Navarro M., Acosta A., Angulo A., Dominguez Z., Robles R., Robles-Zepeda R., Lugo E., Goycoolea F., Velazquez E. (2007). Antibacterial and free-radical scavenging activities of Sonoran propolis. J. Appl. Microbiol..

[42] Silva-Beltrán N.P., Umsza-Guez M.A., Ramos Rodrigues D.M., Gálvez-Ruiz J.C., de Paula Castro T.L., Balderrama-Carmona A.P. (2021). Comparison of the biological potential and chemical composition of Brazilian and Mexican propolis. Appl. Sci..

[43] Vargas-Sánchez R., Torrescano-Urrutia G., Mendoza-Wilson A., Vallejo-Galland B., Acedo-Félix E., SánchezEscalante J., Peñalba-Garmendia M., Sánchez-Escalante A. (2014). Mecanismos involucrados en la actividad antioxidante y antibacteriana del propóleos. Biotecnia.

[44] Ramos A., Miranda J.d. (2007). Propolis: A review of its anti-inflammatory and healing actions. J. Venom. Anim. Toxins Incl. Trop. Dis..

[45] Rufatto L.C., Luchtenberg P., Garcia C., Thomassigny C., Bouttier S., Henriques J.A.P., Roesch-Ely M., Dumas F., Moura S. (2018). Brazilian red propolis: Chemical composition and antibacterial activity determined using bioguided fractionation. Microbiol. Res..

[46] Shukla S.K., Sharma A.K., Gupta V., Yashavarddhan M. (2019). Pharmacological control of inflammation in wound healing. J. Tissue Viability.

[47] González Guevara M.C., Ospina Giraldo L.F., Rincón Velandia J. (2011). Antiinflammatory Activity of Extracts and Fractions of *Myrcianthes leucoxila*, *Calea prunifolia*, *Curatella americana* Y *Physalis peruviana* Obtained from on Tpa-Induced Ear Oedema, Carrageenaninduced Paw Oedema and Collagen-Induced Arthritis. Biosalud.

[48] Gábor M. (2003). Models of acute inflammation in the ear. Inflammation Protocols.

[49] Valenzuela-Barra G., Castro C., Figueroa C., Barriga A., Silva X., de Las Heras B., Hortelano S., Delporte C. (2015). Anti-inflammatory activity and phenolic profile of propolis from two locations in Región Metropolitana de Santiago, Chile. J. Ethnopharmacol..

[50] Guzmán-Gutiérrez S.L., Nieto-Camacho A., Castillo-Arellano J.I., Huerta-Salazar E., Hernández-Pasteur G., Silva-Miranda M., Argüello-Nájera O., Sepúlveda-Robles O., Espitia C.I., Reyes-Chilpa R. (2018). Mexican propolis: A source of antioxidants and anti-inflammatory compounds, and isolation of a novel chalcone and ε-caprolactone derivative. Molecules.

[51] Farooqui T., A Farooqui A. (2010). Molecular mechanism underlying the therapeutic activities of propolis: A critical review. Curr. Nutr. Food Sci..

[52] Blonska M., Bronikowska J., Pietsz G., Czuba Z., Scheller S., Krol W. (2004). Effects of ethanol extract of propolis (EEP) and its flavones on inducible gene expression in J774A. 1 macrophages. J. Ethnopharmacol..

[53] Naito Y., Yasumuro M., Kondou K., Ohara N. (2007). Antiinflammatory effect of topically applied propolis extract in carrageenan-induced rat hind paw edema. Phytother. Res. Int. J. Devoted Pharmacol. Toxicol. Eval. Nat. Prod. Deriv..

[54] Conceição M., Gushiken L.F.S., Aldana-Mejía J.A., Tanimoto M.H., Ferreira M.V.d.S., Alves A.C.M., Miyashita M.N., Bastos J.K., Beserra F.P., Pellizzon C.H. (2022). Histological, Immunohistochemical and Antioxidant Analysis of Skin Wound Healing Influenced by the Topical Application of Brazilian Red Propolis. Antioxidants.

[55] Khorasgani E.M., Karimi A., Nazem M. (2010). A comparison of healing effects of propolis and silver sulfadiazine on full thickness skin wounds in rats. Pak. Vet. J..

[56] Pessolato A.G.T., dos Santos Martins D., Ambrósio C.E., Mançanares C.A.F., de Carvalho A.F. (2011). Propolis and amnion reepithelialise second-degree burns in rats. Burns.

[57] Alberti T., Coelho D., Voytena A., Iacovski R., Mazzarino L., Maraschin M., Veleirinho B. (2019). Effect of propolis nanoparticles on early-stage wound healing in a diabetic noncontractile wound model. Nanotechnol. Adv. Mater. Sci..

[58] Eskandarinia A., Kefayat A., Gharakhloo M., Agheb M., Khodabakhshi D., Khorshidi M., Sheikhmoradi V., Rafienia M., Salehi H. (2020). A propolis enriched polyurethane-hyaluronic acid nanofibrous wound dressing with remarkable antibacterial and wound healing activities. Int. J. Biol. Macromol..

[59] Jaldin-Crespo L., Silva N., Martínez J. (2022). Nanomaterials Based on Honey and Propolis for Wound Healing—A Mini-Review. Nanomaterials.

[60] Kapare H.S., Giram P.S., Raut S.S., Gaikwad H.K., Paiva-Santos A.C. (2023). Formulation Development and Evaluation of Indian Propolis Hydrogel for Wound Healing. Gels.

[61] Khorasani M.Z., Jarrahi M., Jarrahi A. (2016). Combined effect of Iranian propolis and honey on healing of induced incisional wound in rat. IIOAB J..

[62] Marquele-Oliveira F., da Silva Barud H., Torres E.C., Machado R.T.A., Caetano G.F., Leite M.N., Frade M.A.C., Ribeiro S.J., Berretta A.A. (2019). Development, characterization and pre-clinical trials of an innovative wound healing dressing based on propolis (EPP-AF^®^)-containing self-microemulsifying formulation incorporated in biocellulose membranes. Int. J. Biol. Macromol..

[63] Olczyk P., Wisowski G., Komosinska-Vassev K., Stojko J., Klimek K., Olczyk M., Kozma E.M. (2013). Propolis modifies collagen types I and III accumulation in the matrix of burnt tissue. Evid.-Based Complement. Altern. Med..

[64] de Almeida E.B., Cardoso J.C., de Lima A.K., de Oliveira N.L., de Pontes-Filho N.T., Lima S.O., Souza I.C.L., de Albuquerque-Júnior R.L.C. (2013). The incorporation of Brazilian propolis into collagen-based dressing films improves dermal burn healing. J. Ethnopharmacol..

[65] de Albuquerque-Júnior R.L.C., Barreto A.L.S., Pires J.A., Reis F.P., Lima S.O., Ribeiro M.A.G., Cardoso J.C. (2009). Effect of bovine type-I collagen-based films containing red propolis on dermal wound healing in rodent model. Int. J. Morphol..

[66] Stipcevic T., Piljac J., Berghe D.V. (2006). Effect of different flavonoids on collagen synthesis in human fibroblasts. Plant Foods Hum. Nutr..

[67] de Albuquerque R.D.D.G., Perini J.A., Machado D.E., Angeli-Gamba T., dos Santos Esteves R., Santos M.G., Oliveira A.P., Rocha L. (2016). Wound healing activity and chemical standardization of Eugenia pruniformis Cambess. Pharmacogn. Mag..

[68] Houghton P., Hylands P., Mensah A., Hensel A., Deters A. (2005). In vitro tests and ethnopharmacological investigations: Wound healing as an example. J. Ethnopharmacol..

[69] Umachigi S.P., Kumar G., Jayaveera K., Dhanapal R. (2007). Antimicrobial, wound healing and antioxidant activities of Anthocephalus cadamba. Afr. J. Tradit. Complement. Altern. Med..

[70] Alexandru V., Gaspar A., Savin S., Toma A., Tatia R., Gille E. (2015). Phenolic content, antioxidant activity and effect on collagen synthesis of a traditional wound healing polyherbal formula. Stud. Univ. Vasile Goldis Arad. Ser. Stiintele Vietii (Life Sci. Ser.).

[71] Polaka S., Katare P., Pawar B., Vasdev N., Gupta T., Rajpoot K., Sengupta P., Tekade R.K. (2022). Emerging ROS-modulating technologies for augmentation of the wound healing process. ACS Omega.

[72] Wang G., Yang F., Zhou W., Xiao N., Luo M., Tang Z. (2023). The initiation of oxidative stress and therapeutic strategies in wound healing. Biomed. Pharmacother..

[73] Scherer R., Godoy H.T. (2009). Antioxidant activity index (AAI) by the 2,2-diphenyl-1-picrylhydrazyl method. Food Chem..

[74] (2017). Propóleos, Producción y Especificaciones Para su Procesamiento.

[75] Carvalho M.T., Araújo-Filho H.G., Barreto A.S., Quintans-Júnior L.J., Quintans J.S., Barreto R.S. (2021). Wound healing properties of flavonoids: A systematic review highlighting the mechanisms of action. Phytomedicine.

[76] Zulkefli N., Che Zahari C.N.M., Sayuti N.H., Kamarudin A.A., Saad N., Hamezah H.S., Bunawan H., Baharum S.N., Mediani A., Ahmed Q.U. (2023). Flavonoids as potential wound-healing molecules: Emphasis on pathways perspective. Int. J. Mol. Sci..

[77] Nadia Z., Rachid M. (2013). Antioxidant and antibacterial activities of *Thymus vulgaris* L.. Med. Aromat. Plant Res. J..

[78] Kalogeropoulos N., Konteles S.J., Troullidou E., Mourtzinos I., Karathanos V.T. (2009). Chemical composition, antioxidant activity and antimicrobial properties of propolis extracts from Greece and Cyprus. Food Chem..

[79] Fabris S., Bertelle M., Astafyeva O., Gregoris E., Zangrando R., Gambaro A., Lima G.P.P., Stevanato R. (2013). Antioxidant properties and chemical composition relationship of Europeans and Brazilians propolis. Pharmacol. Pharm..

[80] Vargas-Sánchez R., Mendoza-Wilson A., Torrescano-Urrutia G., Sánchez-Escalante A. (2015). Antiradical potential of phenolic compounds fingerprints of propolis extracts: DFT approach. Comput. Theor. Chem..

[81] Rasul A., Millimouno F.M., Ali Eltayb W., Ali M., Li J., Li X. (2013). Pinocembrin: A novel natural compound with versatile pharmacological and biological activities. BioMed Res. Int..

[82] Carballo-Villalobos A., González-Trujano M., López-Muñoz F. (2014). Evidence of mechanism of action of anti-inflammatory/antinociceptive activities of acacetin. Eur. J. Pain.

[83] Fan R., Pan T., Zhu A.-L., Zhang M.-H. (2017). Anti-inflammatory and anti-arthritic properties of naringenin via attenuation of NF-κB and activation of the heme oxygenase (HO)-1/related factor 2 pathway. Pharmacol. Rep..

[84] Nishimura F.d.C.Y., De Almeida A.C., Ratti B.A., Ueda-Nakamura T., Nakamura C.V., Ximenes V.F., Silva S.d.O. (2013). Antioxidant effects of quercetin and naringenin are associated with impaired neutrophil microbicidal activity. Evid.-Based Complement. Altern. Med..

[85] Zhao Y., Liu S. (2021). Bioactivity of naringin and related mechanisms. Die Pharm.-Int. J. Pharm. Sci..

[86] Seelinger G., Merfort I., Schempp C.M. (2008). Anti-oxidant, anti-inflammatory and anti-allergic activities of luteolin. Planta Medica.

[87] Semwal R.B., Semwal D.K., Combrinck S., Trill J., Gibbons S., Viljoen A. (2019). Acacetin—A simple flavone exhibiting diverse pharmacological activities. Phytochem. Lett..

[88] Patel K., Singh G.K., Patel D.K. (2018). A review on pharmacological and analytical aspects of naringenin. Chin. J. Integr. Med..

[89] Özay Y., Güzel S., Yumrutaş Ö., Pehlivanoğlu B., Erdoğdu İ.H., Yildirim Z., Türk B.A., Darcan S. (2019). Wound healing effect of kaempferol in diabetic and nondiabetic rats. J. Surg. Res..

[90] INEGI. Propolis. https://atlas-abejas.agricultura.gob.mx/cap4.html.

[91] Agarwal Y., Beatty C., Ho S., Thurlow L., Das A., Kelly S., Castronova I., Salunke R., Biradar S., Yeshi T. (2020). Development of humanized mouse and rat models with full-thickness human skin and autologous immune cells. Sci. Rep..

[92] Ho A.W., Kupper T.S. (2019). T cells and the skin: From protective immunity to inflammatory skin disorders. Nat. Rev. Immunol..

[93] Florian P., Flechsenhar K.R., Bartnik E., Ding-Pfennigdorff D., Herrmann M., Bryce P.J., Nestle F.O. (2020). Translational drug discovery and development with the use of tissue-relevant biomarkers: Towards more physiological relevance and better prediction of clinical efficacy. Exp. Dermatol..

[94] Leven M., Berghe D.A.V., Mertens F., Vlietinck A., Lammens E. (1979). Screening of higher plants for biological activities I. Antimicrobial activity. Planta Medica.

